# Leveraging Microorganisms to Combat Skin Cancer

**DOI:** 10.3390/microorganisms13020462

**Published:** 2025-02-19

**Authors:** Hayden J. Oyler, Austen W. Callister, Makenzi N. Kutch, Mark R. Wakefield, Yujiang Fang

**Affiliations:** 1 Department of Microbiology, Immunology & Pathology, Des Moines University, West Des Moines, IA 50266, USA; hayden.oyler@dmu.edu; 2 Spencer Fox Eccles School of Medicine, University of Utah, Salt Lake City, UT 84132, USA; austen.callister@hsc.utah.edu; 3 Department of Surgery, School of Medicine, University of Missouri, Columbia, MO 65211, USAwakefieldmr@health.missouri.edu (M.R.W.); 4 Ellis Fischel Cancer Center, School of Medicine, University of Missouri, Columbia, MO 65211, USA

**Keywords:** skin cancer treatment, microorganism cancer treatment, skin microbiota, probiotics, nanocarriers, oncolytic viruses

## Abstract

Skin cancer, including melanoma and non-melanoma types, presents a significant and growing global health challenge due to its increasing incidence and mortality rates. While conventional treatments such as surgical excision, immunotherapy, and targeted therapies are well-established, microorganism-based approaches represent an innovative and promising alternative. These therapies employ live, genetically engineered, or commensal bacteria, viral vectors, or bacterial components to achieve various therapeutic mechanisms, including tumor targeting, immune system modulation, vascular disruption, competitive exclusion, drug delivery, and direct oncolysis. Despite their potential, these approaches require further investigation to address safety concerns, optimize treatment protocols, and gain a comprehensive understanding of their long-term outcomes.

## 1. Introduction

Skin cancer is a common disease worldwide with yearly increasing incidence and mortality rates making it a significant public health concern. According to the GLOBOCAN 2022 data, melanoma and non-melanoma skin cancer (NMSC) are the 17th and 5th most common cancers worldwide, respectively. Melanoma had an estimated 331,722 new cases diagnosed and approximately 58,667 deaths, while NMSC had 1,234,533 new cases and 69,416 deaths. Countries with predominantly fair-skinned populations, including North America, Europe, and Oceania, report the highest incidence rates of melanoma and NMSC, with Australia leading globally [1].

The current skin cancer classifications include three major types: melanoma, basal cell carcinoma (BCC), and squamous cell carcinoma (SCC). Melanoma is characterized by the malignant proliferation of melanocytes and is the third most common skin cancer. It is the most metastatic and has the highest mortality rate among skin cancers [2,3]. BCC, the most common type, arises from basal keratinocytes and rarely metastasizes. SCC originates from keratinocytes and often arises from precancerous lesions called actinic keratosis (AK). It is the second most common type and has a higher metastatic potential than BCC [4]. For this paper, skin cancers will be categorized as either melanoma or NMSC.

Other forms of skin cancer include precancerous lesions and less common cutaneous tumors that fall under the umbrella of skin cancer, such as AK, Merkel cell carcinoma (MCC), and cutaneous lymphomas (CL). AK, a common premalignant lesion, involves dysplasia of epidermal keratinocytes [5]. MCC is a rare, aggressive neuroendocrine carcinoma of the skin that has a high risk of metastasis. CLs encompass a group of lymphoproliferative disorders affecting the skin [6]. While risk factors for skin cancer vary by type, they generally include UV radiation exposure, phenotypic characteristics (e.g., fair skin, light hair), family history, and genetic predisposition [7,8].

Current treatments for skin cancer also depend on the type. For melanoma, common treatments include surgical excision along with adjuvant therapies such as immune checkpoint inhibitors (e.g., pembrolizumab), which have been found to have significantly fewer side effects compared to traditional chemotherapies [9]. In contrast, targeted therapies like *BRAF* inhibitors (e.g., vemurafenib, dabrafenib), which suppress cancer cell proliferation by inhibiting MAPK signaling, are effective but are also known to develop resistance [10]. First-line treatments for BCC and SCC typically involve surgical excision and topical therapies (e.g., imiquimod, 5-fluorouracil) [11]. Standard first-line treatments for both melanoma and NMSC are well-established, but microbial treatments represent a relatively new area of research in skin cancer therapy.

**Figure 1:**
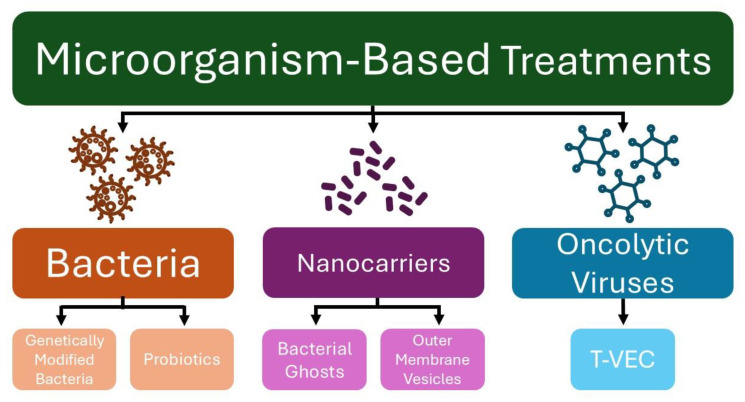
Microorganism-based treatments for skin cancer. (T-VEC—*Talimogene laherparepvec*).

In recent years, microorganisms have been explored as tools for anticancer therapy, serving as vectors for antitumor therapy delivery, vaccines to activate the immune system, and agents to restore or maintain a healthy microbiome (Figure 1) [12]. Although various treatment strategies have been explored to address the rising incidence of skin cancer, the use of microorganisms as therapeutic agents remains relatively underexplored [13]. This paper reviews pre-clinical and clinical studies exploring various microorganism-based treatments for melanoma and NMSC.

## 2. Treatments

### 2.1. Bacterium-Based Therapeutics

Bacterium is known for its ability to accumulate and proliferate in specific areas, making it an ideal candidate for initiating an anti-tumor immune response within the tumor microenvironment. Bacterium-based therapeutics utilize live bacteria to treat cancer and diseases in this fashion. There are different mechanisms in which bacterium-based therapeutics can work, including secreting cytotoxic agents, targeting the microenvironment of the tumor, engineering bacterial vectors to express and release tumoricidal proteins, and manipulating viral and bacterial agents [14]. Bacteria and their products were first used and reported by William Coley, who used two different bacteria strains (*Streptococcus pyogenes* and *Serratia marcescens*) on unresected tumors. The findings showed that the tumor necrosis factor (TNF-α) secretion increased and helped with tumor regression [15].

Bacteria can be engineered by genetic manipulation or synthetic bioengineering for tumor specificity and to specifically create and deliver anticancer agents based on the clinical need [16]. Attenuated *Salmonella typhimurium* is very selective to solid tumors, making it an ideal choice for the treatment of melanoma [17]. The expression of IL-2 allows for an enhanced immune response against a tumor which leads to better suppression of melanoma growth compared to non-cytokine-expressing strains [18]. A different strain of genetically engineered *Salmonella*, AISI-H, uses a dynamic virulence modulation system to heighten its tumor-targeting ability while decreasing off-target toxicity. This specific strain can turn “on” in tumors to allow for the therapeutic effects to take hold and “off” in healthy tissue to avoid unnecessary harm. The AISI-H strain shows exceptional biocompatibility and exhibited notable tumor inhibition and strong immunity activation in a melanoma mouse model [19]. Engineering a non-pathogenic *Escherichia coli* strain has also been used in tumor treatment which releases CD47—a nanobody antagonist that is an anti-phagocytic receptor that is commonly over-expressed in multiple human cancers including melanoma. By using tumor-colonizing bacteria to deliver CD47, it up-regulates the activation of T cells that infiltrate the tumors, triggers expedited tumor regression, and prevents metastasis [20].

Probiotics are bacteria that, when ingested, provide benefits to the host’s health. The idea of using probiotic bacteria in this way was first suggested in 1906 and has since been extensively studied, providing a greater understanding of their various strains, mechanisms, and benefits [21]. They aid the host by maintaining or restoring a healthy balance of gut and skin microbiota and act against pathogenic microorganisms through a variety of mechanisms, including competitive exclusion, mucin and bacteriocin production, and immune system modulation [22]. Probiotic byproducts known as postbiotics are also used as an alternative to whole probiotic administration [23].

In a study investigating the microbiota composition of patients with NMSC, patients with SCC had a higher frequency of *Staphylococcus aureus* than healthy patients in biopsies and swab samples from the skin surface. The study also found a tendency for association of *S. aureus* with AK, but no association with BCC or seborrheic keratosis [24]. *S. aureus* is thought to promote SCC progression by amplifying the immune response and increasing skin inflammation. It does this by secreting phenol-soluble modulin alpha (PSMα) which stimulates keratinocytes to release cytokines IL-1α and IL-36α, creating a cascade to induce the production of the inflammatory cytokine IL-17 [25].

BCC has a distinct microbiota composition compared to other keratinocyte skin tumors such as SCC and AK which are more similar to each other [26]. The most common microbiota in BCC is β-human papillomavirus (HPV) species type 1, while other β-HPV species are found in high frequency in SCC [27]. Certain β-HPV types produce the viral E6 protein which binds to and destabilizes the histone acetyltransferase p300, reducing its ability to activate transcription of the *BRCA1* and *BRCA2* DNA repair genes. Consequently, the cell’s ability to repair DNA double-strand breaks from UV damage is impaired [28]. Furthermore, the E6 protein degrades the pro-apoptotic protein Bak in keratinocytes, as well as destabilizes the tumor suppressor p53 [29,30]. All these mechanisms allow the cell to survive UV damage, accumulate UV-induced mutations, and continue to replicate, adding support to the hypothesis that β-HPV plays a role in progressing skin cancer [28].

There are characteristic bacteria such as *Propionibacterium* spp. found in healthy skin at a higher frequency compared to *S. aureus* in SCC and AK [31]. A healthy population of commensal bacteria on the skin is necessary to maintain eubiosis, often by directly inhibiting pathogen growth and enhancing the host’s innate immunity [32]. *Staphylococcus epidermis* is one of these commensal bacteria species found predominantly on normal human skin [33]. Strains of *S. epidermis* produce 6-N-hydroxyaminopurine (6-HAP) which selectively inhibits the DNA polymerase activity of tumor cells without affecting normal keratinocytes. 6-HAP acts by competing with adenosine to inhibit DNA synthesis, while it is likely multiple mechanisms occur in nature for the inactivation of 6-HAP in the case of normal skin microbiota that remains unaffected [34]. *S. epidermis* has also been shown to stimulate the production of IL-17α+ CD8+ T cells which limit pathogen invasion and strengthen the innate barrier immunity of the epidermis [35]. Lipoteichoic acid (LTA) is a product secreted by *S. epidermis* and other commensal staphylococcal species that increases the production of antimicrobial peptides in mast cells to enhance their infection-fighting ability [36].

In melanoma patients, *Trueperella pyogenes* and *Fusobacterium necrophorum* are among the most common bacteria found in the skin microbiome [37]. The *Fusobacterium* genus is known to sustain inflammation and promote the progression of other cancers, indicated by larger tumors forming more rapidly, and increased proliferation and invasive activity compared to controls [38]. *Fusobacterium* also acts by creating an immunosuppressed environment via interaction between the bacterial Fap2 protein with human T cell immunoglobulin and ITIM domain (TIGIT) to inhibit natural killer cell cytotoxicity [39].

Several studies show that probiotics can be an effective means of treating both NMSC and melanoma. In NMSC, probiotics generally can neutralize pathogenic microbes by stimulating the host’s mucin and bacteriocin secretion to trap pathogens [40]. Treatment of skin with the probiotic bacteria *Lactobacillus reuteri* and *Lactobacillus rhamnosus*, before or concurrent with *S. aureus* infection, showed significant keratinocyte survival. *L. reuteri* and *L. rhamnosus* increased the number of normal primary human epidermal keratinocytes (NHEK) from 8.8% to 53.1% and 42.7%, respectively. *L. reuteri* inhibited *S. aureus* adhesion to keratinocytes by competitive exclusion, independent of inhibitory molecule amplification [41]. Although further study is needed, there is also potential for using *S. epidermis* or its postbiotic 6-HAP as a preventative treatment for skin cancer [34]. In skin tumorigenic mice, treatment with the probiotics *Saccharomyces cerevisiae*, *Bacillus subtilis*, and *Lactobacillus acidophilus* effectively lowered the HPV rate, showing promise for potential treatment of BCC and other NMSCs [42]. Another probiotic, *Lactobacillus salivarius* REN, has been shown to have anti-tumorigenic activity in human tongue SCC, and the probiotic compound AJ2 inhibited the growth of oral cancer lines in humanized mice models [43,44]. To address UV damage, a common factor in NMSC incidence and progression, the postbiotic LTA can be used to treat UV-stressed skin tumors. Produced by *Lactobacillus rhamnosus* GG (LGG), LTA can both prevent formation of UV-induced skin tumors and overcome UV-induced skin immunosuppression [45].

Much of the research conducted on probiotics and melanoma is centered on the effects of probiotics on melanoma immunotherapy [46]. Administration of the probiotic *Bifidobacterium* combined with immunotherapy in a melanoma mouse model was linked to greater tumor outgrowth suppression compared to *Bifidobacterium* administration alone [47]. A different probiotic, *Lactobacillus reuteri* FLRE5K1, was studied for its anti-melanoma activity in cell assays and animal models. The results of the study found that *L. reuteri* FLRE5K1 reduced melanoma incidence by 40% and significantly prolonged survival in tumorigenic mice, likely by stimulating the body’s anti-cancer cytokine production and inhibiting melanoma cell migration [48]. There have also been many studies in recent years exploring the composition of the gut microbiota and understanding how it affects distal tumors. The link between the gut microbiota and melanoma specifically has been well-established, and current research is underway investigating the effectiveness of fecal transplants in melanoma patients [49,50].

The future of bacterium-based therapeutics has many possibilities, especially when considering its use in conjunction with other reliable cancer treatment methods to optimize patient outcomes [51]. Despite the promising potential of bacterium-based therapeutics, however, there are some drawbacks to its use as an agent of tumor-treatment delivery. A primary concern when working with bacteria is that they may trigger a strong immune response in the patient which could result in septic shock. Another potential risk is the body identifying the bacterium as foreign and attacking it, negating the original intent of utilizing bacteria to target the tumors. Additionally, if the bacterium’s targeting-specificity is not honed correctly, it may target and kill healthy cells in the body and trigger early cell death systemically, which can worsen the patient’s prognosis and increase the risk of mortality. Administered bacteria also require correct dosing that varies with the patient; higher dosing can be positively correlated with increased risk of toxicity and decreased anticancer efficacy. These specific concerns, along with the variable stability and consistency of bacterial agents as a whole, create major obstacles for more widespread implementation in cancer treatment [15]. A deeper understanding of the mechanisms employed by these bacteria, along with a more detailed profile of the cancer microbiota, provide promising directions for future research in reducing risks and advancing clinical applications.

### 2.2. Nanocarriers

Engineered nanomaterials called nanocarriers utilize bacterial and other components to enhance drug delivery. Traditional drug delivery methods often face challenges in reaching targeted sites; however, these barriers can be addressed through the selective modification of nanocarriers for cell-specific targeting [52]. Nanotechnology enables the reinforcement of these drug vehicles’ structural integrity to ensure they reach the intended destination, while also allowing for customization of attributes such as size, charge, and composition to meet treatment needs [53]. Biological nanocarriers derived from Archaea and Bacteria have been explored for drug delivery because they enhance cellular uptake, evade the body’s immune response, can fuse to target cells, and are biodegradable [54].

Archaeosomes are a type of nanocarrier made from the phospholipids of various Archaeobacteria. Phospholipids from this domain have several properties that allow them to withstand extreme environmental conditions, making them ideal candidates for drug-delivery systems [55]. Bacterial ghosts (BGs) are empty, Gram-negative cell envelopes that are nonliving and can be loaded with therapeutic agents. BG are desirable for drug delivery because the outer membrane structures are preserved, and they use the body’s inflammatory response and lysozymes to release the drug contents [56]. Outer membrane vesicles (OMVs) are naturally released vesicles from Gram-negative bacteria used for storing and transporting proteins and metabolites [57]. They contain proteases, sulfatases, and adhesins, allowing them to bypass phagocytosis and enter cells by other means [54].

Archaeosomes have been used in treating other cancers and delivering therapeutic cancer vaccines, however, their application in skin cancer is limited [58,59]. BGs, on the other hand, have been shown to have high recognition and internalization by melanoma tumor cells. Results from a study showed that BGs loaded with plasmid DNA were efficiently internalized and phagocytosed by both antigen-presenting cells and tumor cells with 82% of cells expressing the plasmid-encoded reporter gene [60]. BGs have also shown promise as adjuvants to chemotherapy in melanoma [61], while lysate-loaded BGs have been explored as a potential treatment for skin cancer [62]. OMVs show perhaps the greatest success in using nanocarriers for skin cancer treatment. OMVs, derived from transgenic *E. coli* and modified to induce the transdermal photo-TRAIL-programmed treatment in melanoma, exhibited effective penetration of the skin and specificity to melanoma. Furthermore, they successfully induced photothermal–photodynamic responses against primary melanoma spheroids and activated TRAIL-induced apoptosis, resulting in the complete eradication of melanoma with little to no side effects [63]. This finding was reinforced by a previous study of OMVs acting via an interferon-γ-mediated mechanism to induce long-term antitumor responses without apparent adverse effects [64]. Amidst these triumphs, limitations such as toxicity risk and variable efficacy still remain [57]. Although additional research is required, nanocarriers are emerging as effective immunotherapeutic agents with prospects for implementation in clinical trials.

### 2.3. Oncolytic Viruses

Oncolytic viruses (OVs) are viruses, either genetically engineered or naturally occurring, designed to selectively replicate within and destroy cancer cells while sparing normal host tissues [65,66]. Since the mid-1800s, tumor regression has been observed in the presence of incidental viral infections, piquing the interest of researchers and subsequently leading to further investigation of the therapeutic properties of viruses in cancer treatment [67]. OVs combat tumors through three key mechanisms: direct oncolysis, vascular disruption, and immune activation. Through the first mechanism, OVs target and replicate specifically within cancerous endothelial cells, resulting in their destruction while sparing healthy tissue [68]. The second mechanism involves the disruption of tumor vasculature through the delivery of anti-angiogenic genes by oncolytic viruses, which hampers angiogenesis and blood flow to the tumor. Other mechanisms employed to disrupt angiogenesis include targeting the VEGF receptor, E-selectin, and other molecules expressed on endothelial cells especially [69]. This leads to a hypoxic microenvironment, inducing secondary necrotic and apoptotic death in nearby uninfected cells, while amplifying the therapeutic effects of the virus [70]. Finally, OVs stimulate innate and adaptive immune responses by releasing tumor-associated antigens and signals, such as damage-associated molecular patterns (DAMPs) and pathogen-associated molecular patterns (PAMPs). These signals recruit and activate dendritic cells and cytotoxic T cells, enhancing the immune system’s ability to eliminate residual tumor cells [68,71].

The clinical application of OVs is extensive, with melanoma, gastrointestinal, and genitourinary cancers being the most frequently studied in clinical trials [72]. A select number of OVs have been approved for cancer treatment worldwide and have shown significant advantages compared to systemic treatments alone [73]. Talimogene laherparepvec (T-VEC), a genetically modified herpes simplex virus type 1, has been approved in the United States for the treatment of advanced melanoma [65]. Rigvir, an oncolytic virus derived from the ECHO-7 strain of enterovirus, is approved in Latvia, Georgia, Armenia, and Uzbekistan for the treatment of melanoma [74]. H101, a genetically modified adenovirus, has been approved in China as an adjunct to chemotherapy to treat nasopharyngeal carcinoma [75].

Building on their broad clinical applications, oncolytic viruses have demonstrated potential in treating skin cancers, particularly melanoma. The only OV approved for use in the US is T-VEC, which in addition to the aforementioned mechanisms of OVs, has been engineered to be selectively replicated within tumor cells to produce granulocyte–macrophage colony-stimulating factor (GM-CSF) [76]. This induces proliferation, maturation, and migration of dendritic cells, leading to antigen presentation and T cell activation, thus increasing immune response [77]. In the phase III OPTiM trial, T-VEC achieved a durable response rate (DRR) of 16.3% compared to 2.1% with GM-CSF alone and an overall response rate (ORR) of 26.4% versus 5.7% [78,79]. However, in the MASTERKEY-265 trial, T-VEC combined with pembrolizumab (a PD-1 inhibitor used in the treatment of melanoma) failed to show additional clinical benefit compared to pembrolizumab alone [80], thus challenging the efficacy of T-VEC. It should also be noted that while patients with compromised immune systems are at higher risk of developing skin cancer, they are also at higher risk for experiencing adverse effects and complications from T-VEC, considering the fact that it is an active attenuated herpes simplex virus. Complications include herpetic infections, ranging from simple cold sores to severe disseminated infections [81].

The application of OVs for NMSC is currently being explored. One notable study researched the potential use of intralesional T-VEC in patients with cutaneous lymphomas and NMSC, particularly cutaneous SCC and MCC. The results demonstrated a significant reduction of elevation in 84% of tumors and a reduction of redness in 68% of tumors. The non-injected response of tumors observed was 40%, and the overall response in patients treated was 32%, suggesting that T-VEC is a viable treatment option for patients presenting with cutaneous lymphoma and non-melanoma skin cancer [82].

OVs have shown considerable promise as novel treatments for various cancers, including melanoma and NMSC. While the results are promising, further research is needed to optimize treatment protocols, enhance the efficacy of OVs, and understand their long-term effects. Ongoing research is crucial to expanding the therapeutic applications of OVs.

## 3. Conclusions

The rising incidence of skin cancer underscores the need to explore new treatment modalities. While the use of microorganisms in anticancer therapies is still in its early stages, it presents risks that must be considered (Table 1). A primary concern is the potential for infections and their severe consequences, including death. Achieving the desired therapeutic effect requires a balance between stimulating the host’s immune system and attenuating the microorganism’s activity. Additionally, legal challenges arise due to the limited understanding of microbes’ comprehensive impact on cancer [12]. These challenges highlight the critical need for further research in this field. Despite these obstacles, the successes of the therapies discussed in this review emphasize the importance of continued investigation into utilizing microorganisms and their derivatives for treating skin cancer.

## Figures and Tables

**Table 1:** Benefits and risks of different microorganism-based treatments for skin cancer.

Treatment	Benefits	Risks
Bacterium-Based Therapeutics	Preventative	Induce septic shock
	Enhances immune system	Target healthy cells
	Improves multiple body systems	Toxicity from higher dosing
		Variable stability and consistency
Nanocarriers	Reduced side effects	Toxicity
	No reverting to being pathogenic	Variable efficacy
	Highly customizable	
Oncolytic Viruses	High specificity	Herpetic infections
	Preserves healthy tissues	
	Enhances immune system	

## Data Availability

No new data were created or analyzed in this study.

## Abbreviations

The following abbreviations are used in this manuscript:

AK	Actinic Keratosis
BCC	Basal Cell Carcinoma
BG	Bacterial Ghost
CL	Cutaneous Lymphoma
DAMP	Damage-Associated Molecular Pattern
DRR	Durable Response Rate
GM-CSF	Granulocyte–Macrophage Colony-Stimulating Factor
HPV	Human Papillomavirus
LGG	*Lactobacillus rhamnosus* GG
LTA	Lipoteichoic Acid
MCC	Merkel Cell Carcinoma
NMSC	Non-Melanoma Skin Cancer
OMV	Outer Membrane Vesicle
ORR	Overall Response Rate
OV	Oncolytic Virus
PAMP	Pathogen-Associated Molecular Pattern
SCC	Squamous Cell Carcinoma
TIGIT	T Cell Immunoglobulin and ITIM Domain
TNF	Tumor Necrosis Factor
T-VEC	*Talimogene laherparepvec*
